# U-shaped relationship between platelet–lymphocyte ratio and postoperative in-hospital mortality in patients with type A acute aortic dissection

**DOI:** 10.1186/s12872-021-02391-x

**Published:** 2021-11-30

**Authors:** Xi Xie, Xiangjie Fu, Yawen Zhang, Wanting Huang, Lingjin Huang, Ying Deng, Danyang Yan, Run Yao, Ning Li

**Affiliations:** 1 Department of Blood Transfusion, National Clinical Research Center for Geriatric Disorders, Xiangya Hospital, Clinical Transfusion Research Center, Central South University, Hunan Province, 87 Xiangya Road, Changsha, 410008 China; 2 Department of Cardiac Surgery, Xiangya Hospital, Central South University, Hunan Province, Changsha, China; 3 People’s Hospital of Ningxiang, Hunan Province, Ningxiang, China

**Keywords:** Platelet–lymphocyte ratio, Type A acute aortic dissection, In-hospital mortality

## Abstract

**Background:**

The platelet-lymphocyte ratio (PLR), a novel inflammatory marker, is generally associated with increased in-hospital mortality risk. We aimed to investigate the association between PLR and postoperative in-hospital mortality risk in patients with type A acute aortic dissection (AAAD).

**Methods:**

Patients (n = 270) who underwent emergency surgery for AAAD at Xiangya Hospital of Central South University between January 2014 and May 2019 were divided into three PLR-based tertiles. We used multiple regression analyses to evaluate the independent effect of PLR on in-hospital mortality, and smooth curve fitting and a segmented regression model with adjustment of confounding factors to analyze the threshold effect between PLR and in-hospital mortality risk.

**Results:**

The overall postoperative in-hospital mortality was 13.33%. After adjusting for confounders, in-hospital mortality risk in the medium PLR tertile was the lowest (Odds ratio [OR] = 0.20, 95% confidence interval [CI] = 0.06–0.66). We observed a U-shaped relationship between PLR and in-hospital mortality risk after smoothing spline fitting was applied. When PLR < 108, the in-hospital mortality risk increased by 10% per unit decrease in PLR (OR = 0.90, *P* = 0.001). When the PLR was between 108 and 188, the mortality risk was the lowest (OR = 1.02, *P* = 0.288). When PLR > 188, the in-hospital mortality risk increased by 6% per unit increase in PLR (OR = 1.06, *P* = 0.045).

**Conclusions:**

There was a U-shaped relationship between PLR and in-hospital mortality in patients with AAAD, with an optimal PLR range for the lowest in-hospital mortality risk of 108–188. PLR may be a useful preoperative prognostic tool for predicting in-hospital mortality risk in patients with AAAD and can ensure risk stratification and early treatment initiation.

## Background

Type A acute aortic dissection (AAAD) is characterized by an intimal tear based on medial degeneration, with blood surging into the artery wall to form an intimal flap separating the true and false lumen. It is a lethal cardiovascular emergency with a high incidence and early mortality [1, 2]. Predictive biomarkers for identifying increased mortality risk in AAAD patients are important for both risk stratification and early treatment initiation. Inflammation and hemostasis reportedly play pivotal roles in the initiation and progression of AAAD [3, 4]. Inflammatory cells can infiltrate the aortic wall around the vessel and at the margin of the torn media [4, 5]. Moreover, a variety of inflammatory biomarkers have increasingly attracted attention as prognostic indicators in patients with AAAD [6, 7]. Except for inflammatory cells and cytokines, procalcitonin and prognostic nutritional index have also been reported to be related with mortality in AAAD patients [8, 9]. As mediators of inflammation, activated platelets stimulate pathogenic thrombosis in response to atherosclerotic plaque rupture or erosion [10]. Previously, we reported that a low platelet count was a risk factor for postoperative pneumonia in patients with AAAD [11].

The platelet-lymphocyte ratio (PLR) is an integrated reflection of thrombotic/inflammatory pathways. Initially, it was considered a systemic inflammatory biomarker used in the prognosis of neoplastic diseases; however, it is now considered a prognostic marker in cardiovascular diseases [12]. Studies have shown that a high PLR can be used to predict in-hospital mortality in patients with an ST-elevated myocardial infarction [13], without a reflow postpercutaneous intervention [14], and with an acute exacerbation of chronic obstructive pulmonary disease [15]. Few studies have investigated the association between the PLR and the risk of in-hospital mortality in patients with AAAD. Bedel et al. [16] reported that a high PLR at admission was associated with increased in-hospital mortality in patients with AAAD. However, Yang et al. [17] suggested that a high preoperative PLR in patients with AAAD is correlated with a better prognosis. In addition, a study demonstrated that the PLR could be used for the differential diagnoses of similar diseases, but its usefulness in prognosis assessment should be further investigated [18]. Furthermore, there is no existing evidence of a non-linear relationship between PLR and postoperative in-hospital mortality in patients with AAAD.

Therefore, we aimed to explore the relationship between PLR and postoperative in-hospital mortality in patients with AAAD to improve risk stratification and treatment.

## Methods

### Patient selection and study endpoint

In this retrospective cohort study, we identified patients with AAAD who underwent surgery within 48 h of admission to Xiangya Hospital of Central South University between January 2014 and May 2019. Patients with a typical history of chest pain, as well as AAAD-related computed tomography and echocardiography findings, were diagnosed with AAAD. Stanford type A (DeBakey type I and type II) dissection involved the ascending aorta with or without aortic arch. Patients with aches or other related symptoms that occurred within 2 weeks before admission were diagnosed with acute type A dissection [19]. All surgical patients aged ≥ 18 years and diagnosed with AAAD were included. Patients with preoperative severe organ dysfunction or those who died intraoperatively were excluded [20]. Postoperative in-hospital mortality was the primary end point. This study was conducted in accordance with the ethical standards of the Declaration of Helsinki of the World Medical Association and the guidelines for good clinical practice. It was approved by the Medical Ethics Committee of Xiangya Hospital of Central South University (Ethical Number: 2019010038). The requirement for individual consent was waived because of the retrospective nature of the study.

### Surgical procedure

During surgery, the patient lay in the supine position and was administered general anesthesia. The point of incision was the middle of the sternum. Cardiopulmonary bypass (CPB) was performed through the right atrial arteries to the right axillary artery by cannulation. Femoral artery cannulation was performed when the dissection involved the right axillary artery, or the pumping pressure was too high. The right axillary artery was used for intubation, and selective anterograde cerebral perfusion was used for cerebral protection. At moderate hypothermia, the circulation was stopped; the ascending aorta was blocked; and cardiac arrest fluid was perfused. When the nasopharyngeal temperature was 25 °C; the three branch vessels of the aortic arch were blocked; the brain was perfused through the right axillary artery; the aortic arch and the ascending aorta were cut longitudinally; the descending aorta was transected; and the frozen elephant trunk stent was implanted into the true lumen of the distal descending aorta and then released; and the ascending aorta was replaced. When the aortic root or valve was also involved, the Bentall procedure, David procedure, or another procedure was performed simultaneously. The stented graft was then anastomosed end-to-end with the 4-branched vessels. On completion of the anastomosis, perfusion in the lower body was resumed through the perfusion limb of the tetrafurcate graft; the CPB flow was gradually returned to 2.0–2.4 L/m^2^/min; and rewarming was initiated. The branches of the aortic arch were reconstructed during the rewarming phase. Finally, the heartbeat was restored, and the patient’s chest was closed, as performed routinely. Blood pressure during and after operation was measured through the radial and dorsalis pedis arterial line.

### Data collection

We collected patients’ demographic, laboratory, and clinical data by reviewing their medical records. Demographic data included age and sex. Laboratory data included lymphocyte, platelet counts, hemoglobin, international normalized ratio, prothrombin time and activated partial thromboplastin time. Clinical information included perioperative data, smoking history, alcohol consumption, hypertension, diabetes mellitus, Marfan syndrome, hemopericardium, coronary artery disease, cerebrovascular disease, chronic renal failure, and the Penn class. Perioperative data included intraoperative CPB time, ventilator use, operative time, surgery type, autologous blood transfusion (≥ 500 mL), and blood product transfusion (platelet, cryoprecipitate, red blood cells [RBCs], and plasma).

PLR was defined as the platelet to lymphocyte ratio; the platelet and lymphocytes counts were obtained from routine blood tests. The patients’ first venous blood samples taken on admission to the hospital were used to run the routine blood investigations. The ethylenediaminetetraacetic-anticoagulated blood samples were run through the Beckman Coulter LH 750 and Sysmex XN-20A1 analyzers (Beckman Coulter Trading (China) Co., Ltd. Shanghai, China).

### Statistical analysis

Continuous variables were presented as means ± standard deviations (SD) or medians with quartile (Q) ranges. Categorical variables were presented as frequencies and percentages. Continuous variables were compared using the t-test (for a normal distribution) or Mann–Whitney U test (for a skewed distribution). The χ^2^ test was used to compare categorical variables. There was no multicollinearity between independent variables.

**Fig. 1:**
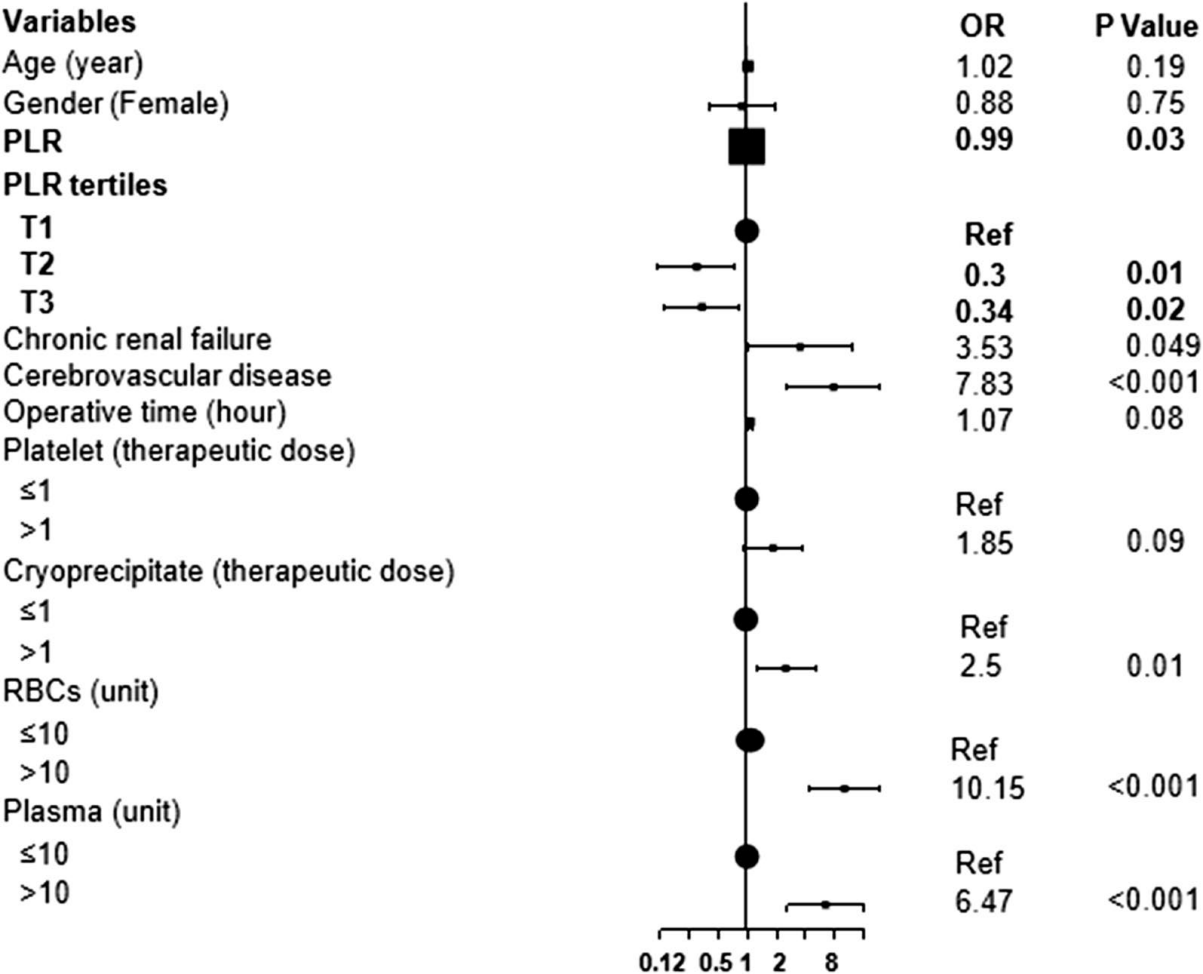
Univariate analysis. Odds ratios and *P* values are presented to show the association between the variables and postoperative in-hospital mortality in patients with AAAD

Based on the PLR, the patients were divided into three tertiles: lowest (T1: PLR < 112.73, n = 90), medium (T2: 112.73 ≤ PLR < 179, n = 90), and highest (T3: PLR ≥ 179, n = 90). The baseline characteristics of the three groups were compared (Table 1). A univariate analysis was used to evaluate the associations between the variables and postoperative in-hospital mortality (Fig. 1). We evaluated the independent effect of PLR on postoperative in-hospital mortality in patients with AAAD using four multiple logistic regression models. No variables were adjusted in Model I, while age and sex were adjusted in Model II. In Model III, we adjusted for age, sex, and variables with *P*-values less than 0.10 (Table 1, Fig. 1). These variables included cerebrovascular disease; chronic renal failure; transfusion of autologous blood (≥ 500 mL), cryoprecipitates, RBCs, plasma, and platelets; surgery type; Marfan syndrome; and operative time. In Model IV, we adjusted for age, sex, and covariances. The covariance adjustment was performed when the variables added to this Model changed the matched odds ratio (OR) by at least 10% [21]. These covariances included alcohol consumption; diabetes mellitus; cerebrovascular disease; chronic renal failure; transfusion of autologous blood (≥ 500 mL), cryoprecipitates, RBCs, plasma, and platelets; and operative time. Smooth curve fitting was performed to determine the linear relationship between PLR and risk of postoperative in-hospital mortality in patients with AAAD. Furthermore, a segmented regression model and the logarithmic likelihood ratio test (LRT) was used to analyze the threshold effect between the PLR and postoperative in-hospital mortality in patients with AAAD.

**Table 1:** Baseline characteristics of patients by PLR tertiles

Variables	PLR tertiles			*P* value
	T1 (< 112.73) (n = 90)	T2 (112.73–179) (n = 90)	T3 (> 179) (n = 90)	
Age (year)	50.93 ± 10.72	49.52 ± 11.65	49.52 ± 11.02	0.618
Sex (male)	69 (76.67%)	57 (63.33%)	63 (70.00%)	0.149
Lymphocyte (10^9^/L)	1.50 (1.20–2.20)	1.20 (1.00–1.40)	0.70 (0.60–1.00)	< 0.001*
Platelet count (10^9^/L)	127.50 (95–150.75)	174 (147.50–215.25)	181 (148.25–232.75)	< 0.001*
Hemoglobin (g/L)	121.70 ± 21.11	122.82 ± 19.07	121.01 ± 22.10	0.840
INR	1.11 (1.04–1.24)	1.09 (1.01–1.17)	1.10 (1.04–1.17)	0.501
PT (s)	14.05 (13.22–15.45)	13.80 (12.93–14.70)	14.00 (13.30–14.90)	0.115
APTT (s)	35.60 (31.85–38.98)	33.55 (29.65–37.95)	33.65 (30.70–37.25)	0.389
Smoking	44 (48.89%)	36 (40.00%)	43 (47.78%)	0.427
Alcohol consumption	31 (34.44%)	32 (35.56%)	33 (36.67%)	0.953
Hypertension	65 (72.22%)	60 (66.67%)	62 (68.89%)	0.719
Diabetes mellitus	3 (3.33%)	6 (6.67%)	4 (4.44%)	0.568
Marfan syndrome	3 (3.33%)	10 (11.11%)	4 (4.44%)	0.067
Hemopericardium	9 (10.00%)	6 (6.67%)	10 (11.11%)	0.564
Coronary artery disease	10 (11.11%)	7 (7.78%)	6 (6.67%)	0.539
Cerebrovascular disease	4 (4.44%)	4 (4.44%)	6 (6.67%)	0.740
Chronic renal failure	5 (5.56%)	3 (3.33%)	4 (4.44%)	0.770
Penn class				0.479
Class Aa	55 (61.11%)	48 (53.33%)	48 (53.33%)	
Non class Aa	35 (38.89%)	42 (46.67%)	42 (46.67%)	
CPB time (min)	176 (146–207.25)	174.5 (143.5–202.75)	176 (144.25–197)	0.424
Ventilator use	51 (56.67%)	46 (51.11%)	51 (56.67%)	0.688
Operative time (hour)	11 (9–12)	10 (9–11.75)	10 (8–12)	0.344
Surgery type				0.055
AAR + TAR (TAVR) + FET	42 (46.67%)	49 (54.44%)	42 (46.67%)	
Bentall + TAR (TAVR) + FET	27 (30.00%)	16 (17.78%)	15 (16.67%)	
David + TAVR + FET	1 (1.11%)	6 (6.67%)	3 (3.33%)	
Combine others	20 (22.22%)	19 (21.11%)	30 (33.33%)	
Autologous blood transfusion (≥ 500 ml)	20 (22.22%)	31 (34.44%)	32 (35.56%)	0.099
Platelet (therapeutic dose)				0.003*
≤ 1	44 (48.89%)	53 (58.89%)	66 (73.33%)	
> 1	46 (51.11%)	37 (41.11%)	24 (26.67%)	
Cryoprecipitate (therapeutic dose)				0.456
≤ 1	53 (58.89%)	58 (64.44%)	61 (67.78%)	
> 1	37 (41.11%)	32 (35.56%)	29 (32.22%)	
RBCs (unit)				0.438
≤ 10	57 (63.33%)	60 (66.67%)	65 (72.22%)	
> 10	33 (36.67%)	30 (33.33%)	25 (27.78%)	
Plasma (unit)				0.099
≤ 10	38 (42.22%)	52 (57.78%)	48 (53.33%)	
> 10	52 (57.78%)	38 (42.22%)	42 (46.67%)	
Postoperative in-hospital mortality	21 (23.33%)	6 (6.67%)	9 (10.00%)	0.002*

Results are expressed as mean ± SD, median (Q1-Q3) or n (%). *, *P* < 0.05. PLR, platelet-lymphocyte ratio; INR, international normalized ratio; PT, prothrombin time; APTT, activated partial thromboplastin time; CPB, cardiopulmonary bypass; AAR, ascending aorta replacement; TAR, total arch replacement; TAVR, total aortic vascular replacement; FET, frozen elephant trunk; RBCs, red blood cells

All statistical analyses were performed with R (http://www.R-project.org, The R Foundation) and EmpowerStats software (http://www.empowerstats.com, X&Y Solutions, Inc, Boston, MA, USA). A two-tailed *P* value < 0.05 was considered significant.

## Results

### Patient characteristics

A total of 280 patients were eligible for this study. After excluding one patient with severe preoperative multiple organ failure (0.37%) and nine patients with intraoperative mortality (3.33%), the remaining 270 patients were enrolled in the study; 70.0% (189/270) were men; and 13.33% (36/270) experienced postoperative in-hospital mortality.

The baseline characteristics are shown in Table 1. There were significant differences in postoperative in-hospital mortality among the tertiles (*P* = 0.002). Intertertile comparisons showed that postoperative in-hospital mortality was the highest, intermediate, and lowest in the T1 (21, 23.33%), T3 (9, 10.00%), and T2 (6, 6.67%) groups, respectively. The number of patients who received platelet transfusion was higher in the T1 and T2 groups (therapeutic dose > 1, *P* = 0.003) than in the T3 group. There were no significant differences in the other variables between the groups.

### Variables related to postoperative in-hospital mortality

The results of the univariate analysis are shown in Fig. 1. PLR and PLR tertiles were negatively associated with the risk of in-hospital mortality (*P* = 0.03). Moreover, chronic renal failure (OR = 3.53, *P* = 0.049); cerebrovascular disease (OR = 7.83, *P* < 0.001); and the transfusion of cryoprecipitates (OR = 2.5, *P* = 0.01), RBCs (OR = 10.15, *P* < 0.001), and plasma (OR = 6.47, *P* < 0.001) were positively associated with the risk of postoperative in-hospital mortality.

### Association of the PLR with postoperative in-hospital mortality

When PLR was used as a continuous variable, it was negatively correlate with postoperative in-hospital mortality in the four models (OR: 0.99, 95% confidence interval [CI]: 0.99–1). When the PLR was used as a tertile categorical variable, the risk of postoperative in-hospital mortality in the T2 group was the lowest in Model I (OR: 0.30, 95% CI: 0.12–0.75), compared to those in the T1 and T3 groups. Similar results were observed in Models II (OR: 0.31, 95% CI: 0.12–0.78), III (OR: 0.17, 95% CI: 0.05–0.58), and IV (OR: 0.2, 95% CI: 0.06–0.66). For the sensitivity analysis, PLR was considered as three equal categorical variables and a continuous variable separately, and the trend of the relationship between PLR and postoperative in-hospital mortality was consistent (*P* < 0.05) (Table 2).

**Table 2:** Multivariable regressions analysis in different models

Variables	Model I (OR, 95% CI)	Model II (OR, 95% CI)	Model III (OR, 95% CI)	Model IV (OR, 95% CI)
PLR	0.99 (0.99, 1.00)*	0.99 (0.99, 1.00) *	0.99 (0.99, 1.00)	0.99 (0.99, 1.00)
PLR tertiles				
T1	Ref	Ref	Ref	Ref
T2	0.30 (0.12, 0.75)*	0.31 (0.12, 0.78) *	0.17 (0.05, 0.58) *	0.20 (0.06, 0.66) *
T3	0.34 (0.14, 0.82)*	0.36 (0.15, 0.87) *	0.32 (0.10, 0.97) *	0.30 (0.09, 0.92) *
*P* value for trend	0.01	0.01	0.02	0.02

*P < 0.05. OR, odds ratio; CI, confidence interval; Ref, reference. Model I, adjusted for none; Model II, adjusted for age and sex; Model III, adjusted for age, sex, cerebrovascular disease, chronic renal failure, autologous blood (≥ 500 ml), cryoprecipitate, RBCs, plasma, platelet, surgery type, Marfan syndrome, operative time; Model IV, adjusted for age, sex, alcohol consumption, diabetes mellitus, cerebrovascular disease, chronic renal failure, autologous blood (≥ 500 ml), cryoprecipitate, RBCs, plasma, platelet, operative time

### Nonlinear relationship between the PLR and risk of postoperative in-hospital mortality

**Fig. 2:**
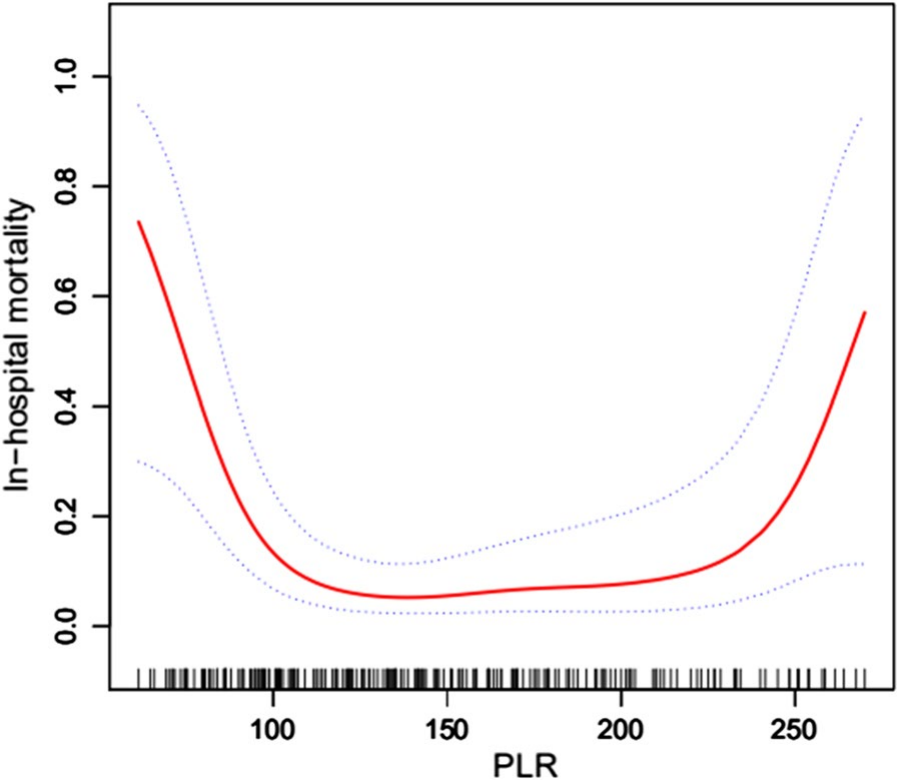
Smoothing spline fitting curve. After adjusting for age; sex; cerebrovascular disease; chronic renal failure; autologous blood (≥ 500 mL), cryoprecipitate, RBCs, plasma, and platelet transfusion; surgery type; Marfan syndrome; and operative time, we observed a U-shaped relationship between PLR and postoperative in-hospital mortality risk in patients with AAAD. Nonlinear plots are displayed with red dotted lines, and the blue dotted lines represent 95% confidence intervals

There was a U-shaped relationship between PLR and postoperative in-hospital mortality risk in patients with AAAD after smoothing spline fitting was applied and covariates were adjusted (Fig. 2). The turning point values of PLR (108, 188) were found using the segmentation regression model (Table 3). When PLR was < 108, the postoperative in-hospital mortality risk increased by 10% per unit decrease in PLR (OR = 0.90, *P* = 0.001). When PLR was between 108 and 188, the mortality risk was the lowest (OR = 1.02, *P* = 0.288). When PLR was > 188, the mortality risk increased by 6% per unit increase in PLR (OR = 1.06, *P* = 0.045). A *P* value < 0.001 for the LRT illustrated a nonlinear relationship between PLR and postoperative in-hospital mortality risk.

**Table 3:** Threshold effect analysis

Models	Adjusted OR (95%CI)	*P*-value
Model I		
One line slope	1.00 (0.99, 1.01)	0.351
Model II		
Turning point (K1, K2)	108, 188	
< 108 slope 1	0.90 (0.85, 0.96)	0.001
108–188 slope 2	1.02 (0.98, 1.05)	0.288
> 188 slope 3	1.06 (1.00, 1.13)	0.045
LRT test	< 0.001^#^	

Data are presented as OR (95% CI) and *P*-value; ^#^ indicates that model II is significant different from model I

Model I, linear analysis; model II, nonlinear analysis. LRT, Logarithmic likelihood ratio test. (*P* < 0.05 means model II is significantly different from model I, which indicates a nonlinear relationship). All adjusted for age, sex, cerebrovascular disease, chronic renal failure, autologous blood (≥ 500 ml), cryoprecipitate, RBCs, plasma, platelet, surgery type, Marfan syndrome, operative time

## Discussion

We observed a U-shaped relationship between PLR and postoperative in-hospital mortality in patients with AAAD and found that 108–188 was the range of optimal PLRs associated with the lowest mortality risk. Moreover, PLR was negatively correlated with postoperative in-hospital mortality risk for PLR < 108 and positively correlated for PLR > 188. Du et al. had similar findings in patients with type B AAD [22]. They observed a U-shaped association between PLR at admission and in-hospital death and indicated that patients with a decreased or elevated PLR had significantly increased in-hospital mortality compared to those with an intermediate PLR. Bedel et al. [16] reported a correlation between high PLR (> 195.8) and increased in-hospital mortality in patients with AAAD. However, Yang et al. [17] found that a high preoperative PLR (≥ 150) was associated with a low incidence of postoperative adverse events. Contrastingly, Sbarouni et al. found no association between the PLR and mortality [18]. The optimal PLR range in our study suggested that maintaining the preoperative PLR within this range might effectively reduce the in-hospital mortality risk.

PLR, as an inflammatory biomarker, positively correlated with adverse prognoses in other diseases. In a study of 520 patients who underwent transcatheter aortic valve replacement, a high PLR was associated with an increased occurrence of 30-day adverse outcomes [23]. Similarly, Ye et al. [24] confirmed a positive relationship between PLR and lower extremity peripheral artery disease severity. Turcato et al. [25] reported that PLR was independently associated with 30-day mortality in patients with acute decompensated heart failure.

The underlying mechanism of the relationship between PLR and mortality in patients with AAAD remains unclear. In our study, a U-shaped relationship existed between PLR and mortality; that is, low PLR (caused by low platelet counts on admission) and high PLR (caused by a low lymphocyte) were related with increased in-hospital mortality risk. Platelets reflected the degree of thrombosis in AAAD, and lower platelet counts suggested platelet dysfunction, which increased the overall bleeding risk of perioperative patients and aggravated ischemia–reperfusion injury [26]. Decreased platelet counts and platelet dysfunction were related to platelet activation [10, 27] and disseminated intravascular coagulation [28]. Activated platelets release inflammatory mediators into the local microenvironment to recruit leukocytes and trigger platelet aggregation [29]. They may deteriorate the state of inflammation and coagulation, aggravate the aortic intimal injury, and increase the incidence of poor outcomes. Furthermore, massive platelet consumption can increase the hazards of dissection rupture and aggravate organ ischemia [10]. Many studies have demonstrated that a low platelet count is associated with increased in-hospital mortality in patients with AAAD [6, 30, 31]. Contrarily, low lymphocyte counts can reflect the severity of inflammation, which is related to poor outcomes in patients with cardiovascular diseases [32]. Physiological stress and overall immune metabolic depression in patients with AAAD can lead to lymphopenia [4, 18], which reduces effectivity against oxidative and inflammatory injury. Taken together, both inflammation and hemostasis play important roles in the pathogenesis and prognosis of AAAD; however, assessment of inflammatory and thrombosis aids in studying the complete thrombo-inflammatory process in AAAD. Based on this mechanism, PLR, as the inflammatory and thrombotic coexistence biomarker, could reflect the degree of thrombosis and inflammation simultaneously and was a stronger predictor of in-hospital mortality than either of those biomarkers alone. Thus, it is reasonable to believe that an elevated or decreased PLR is associated with adverse outcomes in patients with AAAD.

This study had some limitations. First, this was a single-center retrospective study with a small sample size, which may have limited the subgroup analysis and introduced inevitable bias. Furthermore, the lymphocyte cell count was influenced by many factors, including age, gender, race, smoking, pregnancy, infections and so on [33]. However, to control for bias, we adjusted for potential confounding factors during the data analysis; therefore, our results remain credible.

Second, this was a retrospective study and can only show the association between PLR and mortality. Therefore, we will design a prospective, multi-center and large-scale study to verify the U-shaped correlation between PLR and postoperative in-hospital mortality in patients with AAAD. Third, due to the acute onset of AAAD, we could not collect blood samples of patients at the same time of day, this might cause us to ignore the impact of the diurnal or circadian variation of lymphocyte cell count. Fourth, we did not explore the relationship between PLR and the long-term prognosis of patients with AAAD. Currently, these patients have been following up to determine the relationship between PLR and the long-term prognosis.

## Conclusions

We found a novel U-shaped relationship between PLR and postoperative in-hospital mortality in patients with AAAD, with the optimal PLR range for the lowest in-hospital mortality being 108–188. Thus, PLR may serve as a preoperative prognostic tool to predict the risk of in-hospital mortality in patients with AAAD, as this could assist in risk stratification and early treatment initiation.

## Data Availability

The datasets used and analyzed during the current study available from the corresponding authors on reasonable request.

## Abbreviations

AAAD: Type A acute aortic dissection
PLR: Platelet–lymphocyte ratio
CPB: Cardiopulmonary bypass
RBCs: Red blood cells
LRT: Likelihood ratio test
OR: Odds ratio
CI: Confidence interval
